# Knockdown of FOXA1 enhances the osteogenic differentiation of human bone marrow mesenchymal stem cells partly via activation of the ERK1/2 signalling pathway

**DOI:** 10.1186/s13287-022-03133-2

**Published:** 2022-09-05

**Authors:** Lijun Li, Yibo Wang, Zhongxiang Wang, Deting Xue, Chengxin Dai, Xiang Gao, Jianfei Ma, Kai Hang, Zhijun Pan

**Affiliations:** 1 Department of Orthopedics Surgery, The Second Affiliated Hospital, Zhejiang University School of Medicine, Hangzhou City, Zhejiang Province People’s Republic of China; 2 Orthopedics Research Institute of Zhejiang University, Hangzhou City, Zhejiang Province People’s Republic of China; 3 Key Laboratory of Motor System Disease Research and Precision Therapy of Zhejiang Province, Hangzhou City, Zhejiang Province People’s Republic of China; 4 Key Laboratory of Image Information Processing and Intelligent Control, School of Artificial Intelligence and Automation, Huazhong University of Science and Technology, Wuhan, People’s Republic of China; 5 Clinical Research Center of Motor System Disease of Zhejiang Province, Zhejiang Province Hangzhou City, People’s Republic of China

**Keywords:** Osteogenic differentiation, FOXA1, Bioinformatics, ERK1/2

## Abstract

**Background:**

The available therapeutic options for large bone defects remain extremely limited, requiring new strategies to accelerate bone healing. Genetically modified bone mesenchymal stem cells (BMSCs) with enhanced osteogenic capacity are recognised as one of the most promising treatments for bone defects.

**Methods:**

We performed differential expression analysis of miRNAs between human BMSCs (hBMSCs) and human dental pulp stem cells (hDPSCs) to identify osteogenic differentiation-related microRNAs (miRNAs). Furthermore, we identified shared osteogenic differentiation-related miRNAs and constructed an miRNA-transcription network. The Forkhead box protein A1 (FOXA1) knockdown strategy with a lentiviral vector was used to explore the role of FOXA1 in the osteogenic differentiation of MSCs. Cell Counting Kit-8 was used to determine the effect of the knockdown of FOXA1 on hBMSC proliferation; real-time quantitative reverse transcription PCR (qRT-PCR) and western blotting were used to investigate target genes and proteins; and alkaline phosphatase (ALP) staining and Alizarin Red staining (ARS) were used to assess ALP activity and mineral deposition, respectively. Finally, a mouse model of femoral defects was established in vivo, and histological evaluation and radiographic analysis were performed to verify the therapeutic effects of FOXA1 knockdown on bone healing.

**Results:**

We identified 22 shared and differentially expressed miRNAs between hDPSC and hBMSC, 19 of which were downregulated in osteogenically induced samples. The miRNA-transcription factor interaction network showed that FOXA1 is the most significant and novel osteogenic differentiation biomarker among more than 300 transcription factors that is directly targeted by 12 miRNAs. FOXA1 knockdown significantly promoted hBMSC osteo-specific genes and increased mineral deposits in vitro. In addition, p-ERK1/2 levels were upregulated by FOXA1 silencing. Moreover, the increased osteogenic differentiation of FOXA1 knockdown hBMSCs was partially rescued by the addition of ERK1/2 signalling inhibitors. In a mouse model of femoral defects, a sheet of FOXA1-silencing BMSCs improved bone healing, as detected by microcomputed tomography and histological evaluation.

**Conclusion:**

These findings collectively demonstrate that FOXA1 silencing promotes the osteogenic differentiation of BMSCs via the ERK1/2 signalling pathway, and silencing FOXA1 in vivo effectively promotes bone healing, suggesting that FOXA1 may be a novel target for bone healing.

## Background

As one of the most common complications following severe fracture, bone tumour ablation, and debridement of a variety of bone infections, large bone defects continue to be a challenge for orthopaedic surgeons [1, 2]. Numerous studies have demonstrated that the complications linked to large bone defects are closely related to the osteogenic differentiation of bone mesenchymal stem cells (BMSCs) [3–6]. Due to bone graft-associated donor site morbidity**,** tissue engineering research has investigated BMSCs for several years as one of the body's own mechanisms for bone repair [7, 8]. BMSCs possess self-renewal capabilities and the ability to differentiate into a variety of cell types, such as osteoblasts, chondrocytes, and adipocytes [9, 10], which play a critical role in bone formation and are regulated by genetic factors [11]. Furthermore, gene-modified BMSCs could be a promising therapy for bone healing by enhancing osteogenic differentiation of BMSCs [5, 12–17].

In bioinformatics, *high-throughput sequencing* is a novel technique that plays an important role in the exploration of genome‐level differences and provides valuable insights for the identification of key genes and functional pathways associated with osteogenic differentiation in stem cells [18]. BMSCs and DPSCs show similar morphology, proliferative ability, surface marker profiles, and trilineage-differentiation potential in vitro [19–22]. Bone remodelling and regeneration are highly regulated, multistep processes involving post-transcriptional regulation by miRNAs and transcription factors (TFs) [23, 24]. Furthermore, it is becoming increasingly evident that miRNAs regulate the expression of many transcription factors [25].

To identify osteogenic differentiation-related miRNAs, we performed differential expression analysis of miRNAs between undifferentiated stem cells and osteogenically induced samples for hDPSCs and human BMSCs (hBMSCs). Furthermore, we identified shared osteogenic differentiation-related miRNAs and constructed an miRNA-transcription network. Among the transcription factors, Forkhead box protein A1 (FOXA1) has been identified as a novel and crucial osteogenic differentiation biomarker directly targeted by 12 miRNAs.

FOXA1, previously designated hepatocyte nuclear factor 3α is a member of the Fox family of transcription factors involved in the regulation of cell proliferation, development, differentiation, metabolism, and ageing [26–32]. Compared to normal tissue, higher expression of FOXA1 is commonly detected in tumours arising from organs such as the liver, kidney, pancreas, lung, prostate, and mammary glands [33–35]. Furthermore, the Fox family of transcription factors is tightly associated with bone metabolism [36] and a variety of bone diseases, such as osteoarthritis [37], rheumatoid arthritis [38], osteoporosis [29, 39], intervertebral disc degeneration [40], and bone tumours [41]. Overexpression of FOXC2 acts on the Wnt signalling pathway to promote the osteogenic differentiation of BMSCs [42, 43]. Knockdown of FOXF1 also promotes BMSC osteogenesis, in part, by activating the Wnt/β-catenin signalling pathway [39]. Additionally, FOXA2 promotes prostate cancer growth in bone [44].

Despite this current understanding, little is known about the role of FOXA1 in the osteogenic differentiation of hBMSCs. In this study, we address this gap using bioinformatics analysis and in vitro experimental verification. These results indicate that knockdown of FOXA1 promotes osteogenic differentiation of hBMSCs, partly via activation of the ERK1/2 signalling pathway. Furthermore, using a mouse model of femoral defects, we showed that the knockdown of FOXA1 in BMSCs accelerated bone healing in vivo, suggesting that FOXA1 could be a novel target for bone healing.

## Methods

### Data collection

miRNA expression profiles of osteogenic differentiation-related datasets were collected from the Gene Expression Omnibus database (GEO). Two datasets, GSE138180 and GSE107279, were downloaded for further analyses. Dataset GSE138180 is associated with the osteogenic differentiation of hDPSCs and includes miRNA expression profiles of six samples, in which three samples were undifferentiated and the other three were osteogenically induced at day 14. Dataset GSE107279 is associated with the osteogenic differentiation of hBMSCs and includes three undifferentiated samples and three osteogenically induced samples on day 14. In this study, hDPSC and hBMSC samples were undifferentiated and regarded as the control group, and osteogenically induced samples were the experimental group.

### Differential expression analysis

We independently performed differential expression analysis between the undifferentiated samples and the osteogenically induced hDPSCs and hBMSCs. The R package ‘limma’ was used to identify differentially expressed miRNAs. The normalised expression profiles of miRNAs in the control and experimental groups were inputted into the ‘limma’ package, and differentially expressed miRNAs were identified based on two criteria: (1) logFC and (2) BH-adjusted *p* value. An miRNA was identified as upregulated if logFC > 1 and the BH-adjusted *p* value < 0.05, and downregulated if logFC < − 1 and the BH-adjusted *p* value < 0.05. A volcano plot was generated using the ‘ggplot’ R package.

### Construction of miRNA-transcription network

Based on the differential expression analysis of hDPSCs and hBMSCs, 22 shared osteogenic differentiation-related miRNAs were identified. To construct the miRNA-transcription factor interaction network of these 22 miRNAs, we collected transcription factor interaction data from the TransmiR database. This not only includes data from not only published literature but also computational tools. We first downloaded the interaction data, and more than 300 transcription factors were identified as targets of these miRNAs. The miRNA-transcription factor interaction network was plotted using Cytoscape software.

### Cell culture, reagents, and antibodies

HBMSCs and mBMSCs were provided by Cyagen Biosciences (HUXMA-01001, MUBMX-01001, Guangzhou, China), which can differentiate into osteoblasts, chondrocytes, and adipocytes under specific inductive conditions. Adherent hBMSCs were incubated in culture flasks in a special complete growth medium (HUXMA-90011, Cyagen Biosciences, Inc., Guangzhou, China) in a cell incubator at 37 °C with 5% CO_2_ and were passaged at nearly 80–90% confluence. Cells from passages two to six were used in subsequent experiments.

Specific antibodies against glyceraldehyde-3-phosphate dehydrogenase (GAPDH) and FOXA1 were purchased from PROTEINTECH (Chicago, USA). Runt-related transcription factor 2 (RUNX2), extracellular signal-regulated kinase 1/2 (ERK1/2), phospho-ERK1/2 (p-ERK1/2) were obtained from Cell Signalling Technology (Danvers, MA, USA). Specific antibodies against collagen type I alpha 1 (COL1A1) and Osterix (SP7) were purchased from Abcam (Cambridge, UK) and PROTEINTECH (Chicago, USA). A phospho-p44/42 MAPK (P-ERK1/2) inhibitor, PD98059, was purchased from MedChemExpress (New Jersey, USA).

### Lentiviral packaging and cell infection

A lentiviral package was applied by Obio Technology (Shanghai, China) for the hBMSCs, including lentiviral particles to knockdown FOXA1 (FOXA1 knockdown group, KD), knockdown control particles (FOXA1 KD negative control group, KD-NC), lentiviral particles to overexpress FOXA1 (FOXA1 overexpress group, OE) and overexpress control particles (FOXA1 OE negative control group, OE-NC). When hBMSCs reached 30–50% confluence, they were grown in lentiviral particles with 5 ug/ml polybrene in the growth medium according to the manufacturer’s instructions. The fluorescence of GFP was used to determine the transduction efficiency, and the culture medium was changed after 12 h. After three days, the cells were screened with puromycin (2 g/mL) and passaged for use in subsequent experiments. FOXA1 expression was measured using quantitative real-time PCR (qRT-PCR) and western blot analysis.

The lentiviral package for mouse BMSCs (mBMSCs) was applied by GeneChem (Shanghai, China), and the mBMSCs were infected by incubating them in growth media with lentiviral particles and polybrene (5 g/mL). The infection medium was removed after approximately 24 h. After three days, the cells were puromycin (4 g/mL) screened and passaged for use in subsequent investigations. GFP fluorescence was used to measure the transduction efficiency.

### Osteogenic differentiation protocol

HBMSCs were cultured in special growth medium (Cyagen Biosciences, Guangzhou, China) and 100 IU/ml penicillin/streptomycin in six or twelve well cell culture plates (Kangning, Shanghai, China) and incubated for 48 h at 37 °C with 5% CO_2_. Subsequently, the cells were cultured in osteogenic induction medium (HUXMX-90021, Cyagen Biosciences, Inc., Guangzhou, China). The cells were maintained by replacing the osteogenic induction medium with fresh medium every three days.

### RNA extraction and quantitative RT-PCR

Gene expression levels were measured using qRT-PCR. RNAiso reagent (AG, Shanghai, China) and NanoDrop 2000 were used to isolate and measure total cellular RNA. The absorbance of the samples at 260 nm was measured according to the manufacturer’s instructions (Thermo Fisher Scientific). In a 10-µL reaction volume, total RNA was reverse transcribed into complementary DNA (cDNA). Next, 1 ul of cDNA was used as the template with Power SYBR® Green PCR Master Mix (AG, Shanghai, China) and the ABI 7500 System (Thermo Fisher Scientific) was used to perform qRT-PCR in triplicate. 18S rRNA or GAPDH was used as a housekeeping gene, and each reaction was independently repeated three times. Primers were synthesised by Tsingke Biotechnology (Hangzhou, China), and the primer sequences used are listed in Table 1. The qRT-PCR reaction was performed at 95 °C for 30 s, followed by 45 cycles at 95 °C for 5 s and 60 °C for 30 s. The expression levels of all genes were evaluated using the 2^−△△Ct^ method.

**Table 1:** Sequences of primers for real-time quantitative PCR analysis

	Primer sequence, 5′–3′	
Gene	Forward	Reverse
FOXA1	TCCAGGATGTTAGGAACTGTG	AGGCCTGAGTTCATGTTGCT
18S	CGCCGCTAGAGGTGAAATTC	TTGGCAAATGCTTTCGCTC
COL1A1	CAGATCACGTCATCGCAC AAC	GAGGGCCAAGACGAAGAC ATC
OPN	CTCCATTGACTCGAACGA CTC	CAGGTCTGCGAAACTTCT TAGAT
RUNX2	ACTTCCTGTGCTCGGTGCT	GACGGTTATGGTCAAGGTGAA
SP7	AGCCCATTAGTGCTTGTAAAGG	CCTCTGCGGGACTCAACAAC

### Western blotting analysis

Cells were extracted in six-well plates by lysis for 30 min on ice in RIPA buffer containing phosphatase and protease inhibitor cocktails (BOSTER, Wuhan, China). Centrifugation (Eppendorf, 5424R, *Germany*) to clear the lysates and collect the supernatants was performed at 14,000 rpm for 12 min at 4 °C. Equal amounts of protein were separated by 10% sodium dodecyl sulphate polyacrylamide gel electrophoresis and then transferred to a polyvinylidene fluoride membrane (Millipore, Shanghai, China). The membranes were then blocked for 1 h at room temperature in Tris-buffered saline containing 10% non-fat milk or 5% bovine serum albumin (BSA) and 0.1% Tween. Subsequently, the membranes were incubated with primary antibodies overnight at 4 °C. After washing with 0.1% Tween in tris-buffered saline three times every 10 min and incubating with horseradish peroxidase-conjugated secondary antibodies (anti-rabbit; BOSTER) for 1 h at room temperature, proteins were visualised using an enhanced chemiluminescent detection reagent (Millipore) and an XRS chemiluminescence detection system (Bio-Rad Laboratories, Hercules, CA, USA).

### ALP staining

For ALP staining, cells cultured in osteogenic induction medium for 7 d in 24-well plates were fixed with 4% paraformaldehyde (Beyotime, Shanghai, China) for 20–30 min at room temperature and rinsed three times with phosphate-buffered saline (PBS). The cells were stained using a BCIP/NBT ALP colour development kit (Beyotime, Shanghai, China). An ALP activity assay (Beyotime) was used to determine ALP activity according to the manufacturer’s instructions.

### ARS staining

HBMSCs were passaged in 24-well plates and cultured in an osteogenic induction medium for 14 d. Cells were fixed in 4% paraformaldehyde (Beyotime, Shanghai, China) for 20–30 min at room temperature and then rinsed three times with PBS. Alizarin Red S solution (Beyotime) was then added and incubated at room temperature for 15 min followed by rinsing with PBS three times. ARS quantification was performed as previously described [45]. The stain was incubated with 10% cetylpyridinium chloride for 1 h at room temperature, and the solutions were collected and plated in a 96-well plate and read at 560 nm with a microplate reader (ELX808; BioTek). The results were normalised to those of the control group.

### Immunofluorescence

HBMSCs were cultured in a 24-well plate, and RUNX2 and COL1A1 were detected using a fluorescence microscope (EU5888; Leica, Wetzlar, Germany). The cells were treated with 4% paraformaldehyde at room temperature. After 15 min, the cells were permeabilised for 30 min with 0.1 per cent Triton X-100 in PBS and blocked for 30 min with 2% bovine serum albumin (BSA). After washing three times, the fixed cells were incubated at 4 °C overnight with anti-RUNX2 (1:6400; CST) and anti-COL1A1 antibodies (1:500; PROTEINTECH). The cells were then incubated for 60 min at room temperature with a fluorescence-conjugated secondary antibody (Beyotime), and the nuclei were stained for 5 min with DAPI (Beyotime).

### Cell Counting Kit-8

Cells were seeded into 96-well plates with growth medium (Cyagen) at a density of 5000 cells. Ten per cent CCK-8 (Beyotime) was then added to the wells, and the cells were incubated for 2 h at 37 °C. The cell proliferation was evaluated using a microplate reader (ELX808; BioTek, USA) at 450 nm.

### Cell sheet preparation

The infected cell sheet production process followed previously published protocols [46–48]. Brefily, confluent cells (1 × 10^5^/cm^2^) were grown in flasks for 2 weeks in MSC growth medium with vitamin C (20 µg/mL) to generate a sheet of mBMSCs. The cells were then rinsed three times with PBS before being scraped from the intact substratum as cell sheets.

### In vivo* evaluation in animals*

All C57/B16 mice (male, 12 weeks old) were supplied by the Zhejiang Center of Laboratory Animals. All animal experiments were approved by the Institutional Animal Care and Use Committee of Zhejiang Center of Laboratory Animals (ZJCLA-IACUC-20030051).

Thirty male C57/B16 mice were used to establish a mouse model of femoral defects. All mice were divided randomly and evenly into three groups: (1) control group, (2) KD-NC (negative control group of mBMSCs with FOXA1-KD), and (3) KD (mBMSCs with FOXA1-KD) (*n* = 10 per group). The mouse defect model was constructed as previously reported [49–52], and the surgical operations were performed by two experienced orthopaedic surgeons. Briefly, mice were anaesthetised by intraperitoneal injection of 0.3% pentobarbital sodium (30 mg/kg body weight). A skin incision was made over the right lateral femoral aspect and the quadriceps was bluntly dissected to expose the femoral diaphysis. Using a hollow drill, perforations with diameters of 1 mm were created locally. To remove the bone pieces from the cavity, the holes were washed with PBS. As previously mentioned, mBMSCs sheets with FOXA1-KD and negative controls were cultured in flasks for 2 weeks with confluent cells (1 × 10^5^/cm^2^). The details of the cell sheet implantation have been previously described [46, 48]. Briefly, nothing was grafted onto the tibial defect site in the blank group (*n* = 10), and in the KD-NC group, sheets of KD-NC mBMSCs were used to fill the defects and wrap the defect areas. In the KD group, sheets of mBMSCs with FOXA1-KD were implanted into the defects and used to wrap the defect areas. The muscles were then realigned, and the skin was stitched. Two and four weeks after surgery, mice were killed in a CO_2_ container, and specimens of experimental and contralateral intact tibias were collected for subsequent experiments.

#### Radiographic analysis

Following harvest, 4 weeks of samples (*n* = 5 for each group) were sent for microcomputed tomography (μCT) evaluation. Each tibia was scanned using a U-CT system (MILabsU-CT, Netherlands), and the operation parameters were based on a previous report [53].

#### Histological evaluation

Following harvest, the 2-week samples (*n* = 5 for each group) were fixed with 4% paraformaldehyde (Beyotime) for 36 h at 4 °C and then decalcified with ethylene diaminetetra acetic acid decalcifying solution (E1171, Salarbio, Beijing, China) for 1 week at room temperature. The specimens were then embedded in paraffin using standard procedures. Serial Sects. (4 µm thickness) were cut, mounted onto polylysine-coated glass slides, and de-paraffinised. The sections were then stained with HE, Safranin O/Fast Green, and Masson’s trichrome staining were performed separately in consecutive tissue sections in accordance with previous studies [45, 48].

### Statistical analysis

GraphPad Prism v.7.0 was used for all statistical analyses (GraphPad Software, USA). All experiments were performed at least in triplicate. The data are presented as the mean ± SD. Statistical significance was determined by a two-tailed Student's *t* test when comparing two groups, and one-way ANOVA followed by Tukey’s post hoc test when comparing more than two groups. A *p* value of ≤ 0.05 was considered to indicate statistical significance.

## Results

### Differentially expressed miRNAs during the osteogenic differentiation of hDPSCs and hBMSCs

**Fig. 1:**
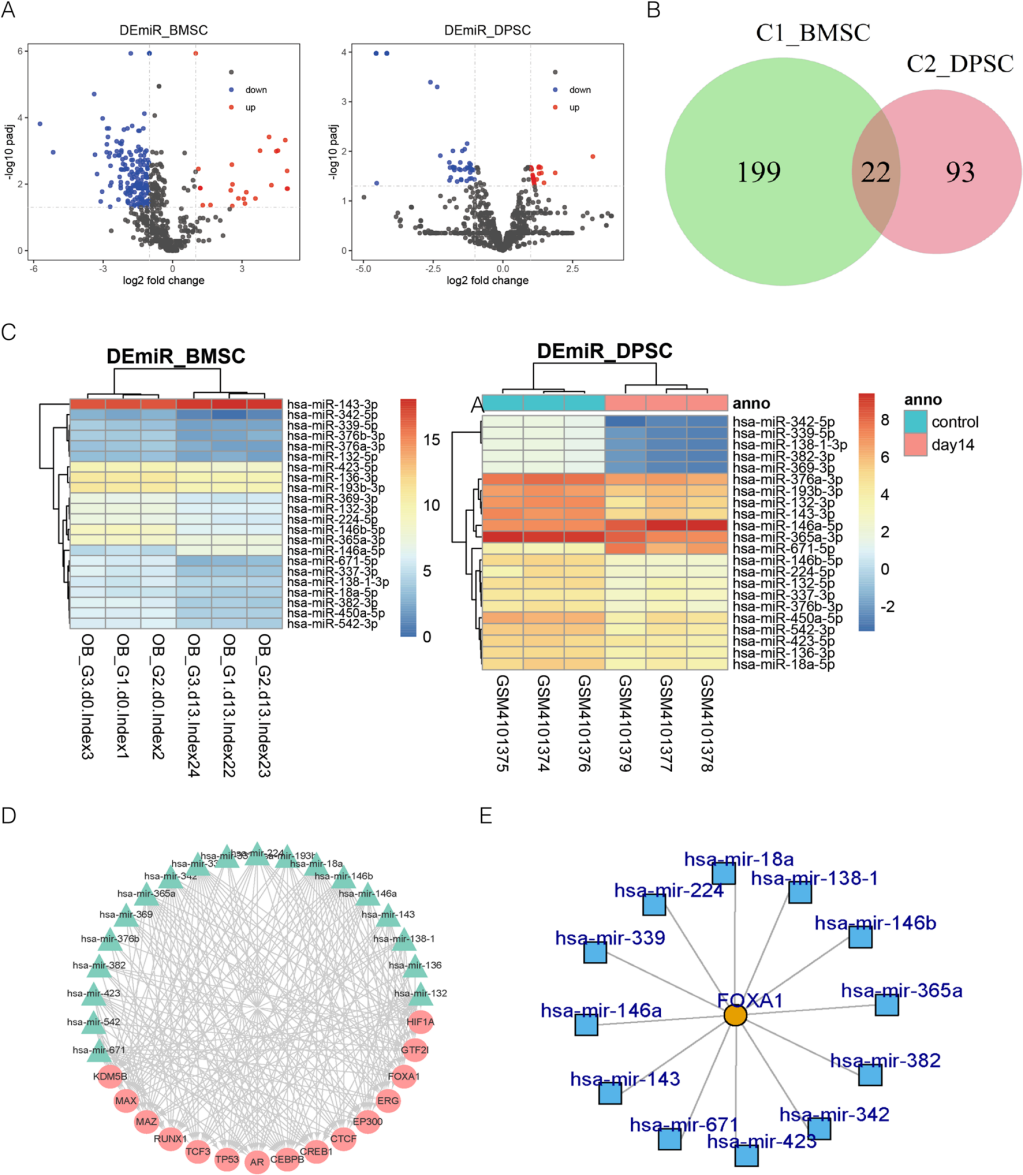
FOXA1 is identified as the novel osteogenic differentiation biomarker. **A** Volcano plot shows differentially expressed miRNAs between undifferentiated samples and osteogenically induced samples in hDPSCs and hBMSCs. **B** Venn diagram shows the intersection of differentially expressed miRNAs derived from hDPSCs and hBMSCs. **C** Heatmap shows the expression of 22 candidate miRNAs between control and osteogenically induced samples in hBMSCs and hDPSCs. **D** Interaction network between transcription factors and miRNAs among these candidate miRNAs. **E** Subgraph of transcription factor-miRNA interaction network of FOXA1

To identify osteogenic differentiation-related miRNAs, we performed differential expression analysis for two datasets, GSE138180 and GSE107279, which are associated with osteogenesis in hDPSCs and hBMSCs, respectively. We found that 221 miRNAs were differentially expressed between undifferentiated samples and osteogenically induced samples, of which 195 were downregulated and 25 were upregulated (Fig. 1A). The analysis of the hDPSCs showed that 115 miRNAs were differentially expressed, of which 27 were upregulated and 88 were downregulated in the osteogenically induced samples (Fig. 1A). These results indicate that a large number of miRNAs contribute to osteogenic differentiation.

### Shared epigenetic regulation of miRNA between hDPSCs and hBMSCs during osteogenic differentiation

We aimed to determine whether miRNAs contribute equally to osteogenic differentiation across different tissues. We compared miRNAs between hDPSCs and hBMSCs to identify those miRNAs that are differentially expressed during osteogenic differentiation. The results showed 22 shared miRNAs (Fig. 1B), and 199 and 93 miRNAs contributed independently to the osteogenic differentiation of hBMSCs and hDPSCs, respectively.

Among all the shared differentially expressed miRNAs, most were found to display the same expression pattern in which 19 miRNAs were downregulated in the osteogenically induced samples including hsa-miR-450a-5p, hsa-miR-132-3p, hsa-miR-146b-5p, and hsa-miR-136-3p; and one miRNA hsa-miR-146a-5p was upregulated during osteogenic differentiation (Fig. 1C). This suggests that the downregulation of these miRNAs can promote osteogenic differentiation by indirectly and epigenetically activating ossification-related targets. In addition to these miRNAs, two miRNAs (hsa-miR-671-5p and hsa-miR-143-3p) displayed different expression patterns between hDPSCs and hBMSCs. All of these miRNAs are listed in Table 2. Collectively, these results suggest that these shared miRNAs are significant regulators in mediating osteogenic differentiation by indirectly activating their shared targets.

**Table 2:** Shared osteogenic differentiation-related miRNAs that are differentially expressed in both BMSC and DPSC

miRNA	Bmsc			DPSC		
	LogFC	Adj.P.val	Regulation	LogFC	Adj.P.val	Regulation
hsa-miR-450a-5p	− 1.744	0.000177	Down	− 1.772	0.021928	Down
hsa-miR-132-3p	− 1.263	0.000211	Down	− 1.869	0.033694	Down
hsa-miR-146b-5p	− 2.829	0.000211	Down	− 2.597	0.000404	Down
hsa-miR-136-3p	− 1.131	0.000984	Down	− 1.250	0.021928	Down
hsa-miR-671-5p	− 2.845	0.0011	Down	3.225	0.012727	Up
hsa-miR-337-3p	− 2.140	0.002462	Down	− 1.294	0.039357	Down
hsa-miR-146a-5p	2.561	0.002592	Up	1.876	0.027187	Up
hsa-miR-193b-3p	− 1.234	0.002646	Down	− 1.018	0.036823	Down
hsa-miR-382-3p	− 2.055	0.002646	Down	− 4.159	0.000106	Down
hsa-miR-143-3p	1.114	0.003485	Up	− 1.792	0.009789	Down
hsa-miR-369-3p	− 1.362	0.003657	Down	− 4.159	0.000106	Down
hsa-miR-365a-3p	− 1.151	0.004975	Down	− 1.639	0.040561	Down
hsa-miR-138–1-3p	− 1.108	0.006419	Down	− 4.159	0.000106	Down
hsa-miR-542-3p	− 1.503	0.007007	Down	− 1.489	0.037778	Down
hsa-miR-339-5p	− 1.534	0.00922	Down	− 4.159	0.000106	Down
hsa-miR-376b-3p	− 1.621	0.011005	Down	− 1.761	0.021225	Down
hsa-miR-376a-3p	− 1.493	0.01275	Down	− 1.177	0.021272	Down
hsa-miR-423-5p	− 1.284	0.013206	Down	− 1.131	0.035079	Down
hsa-miR-132-5p	− 1.065	0.013524	Down	− 1.897	0.021225	Down
hsa-miR-342-5p	− 1.978	0.01995	Down	− 4.542	0.000106	Down
hsa-miR-18a-5p	− 1.215	0.021951	Down	− 1.527	0.016776	Down
hsa-miR-224-5p	− 1.188	0.033804	Down	− 1.914	0.038533	Down

### Osteogenic differentiation-related transcription factors

To identify osteogenic differentiation-related transcription factors, we constructed an miRNA-transcription factor interaction network. We found that more than 300 transcription factors were targeted by these miRNAs. The top 15 transcription factors (HIF1A, GTF2I, FOXA1, ERG, EP300, CTCF, CREB1, CEBPB, AR, TP53, TCF3, RUNX1, MAZ, MAX, and KDM5B) and their corresponding miRNAs are shown in Fig. 1D. A literature search for these transcription factors showed that almost all of them are associated with osteogenic differentiation. HIF1A enhances the osteogenesis of BMSCs in vitro [54]; GTF2I knockdown has a negative effect on the osteogenic differentiation of MSCs [55]; there is overwhelming evidence supporting the notion that ERG plays essential roles in osteogenesis and bone development [56–58]; EP300 is found to be associated with distraction-induced osteogenesis of stem cells [59]; CEBPB has been demonstrated to be a crucial cofactor in the promotion of osteogenic differentiation as a key regulator for RUNX2 [60–62]; va the androgens receptor (AR), androgens regulate bone matrix production, organisation, and mineralisation by enhancing osteoblast differentiation [63–66]; TP53, a widely studied tumour protein, was recently found to promote osteogenic differentiation of BMSC by targeting Smad7 [67]; RUNX1 has been reported to regulate osteogenic differentiation by inhibiting adipogenesis through the Wnt/β-catenin pathway [68]; however, the role of FOXA1, which is directly targeted by 12 miRNAs in the osteogenic differentiation of hBMSCs, is unclear, suggesting that FOXA1 is a potential osteogenic differentiation-related biomarker (Fig. 1E). A similar result was reported in a previous study [69].

### Establishment of FOXA1 knockdown and overexpression in BMSCs

**Fig. 2:**
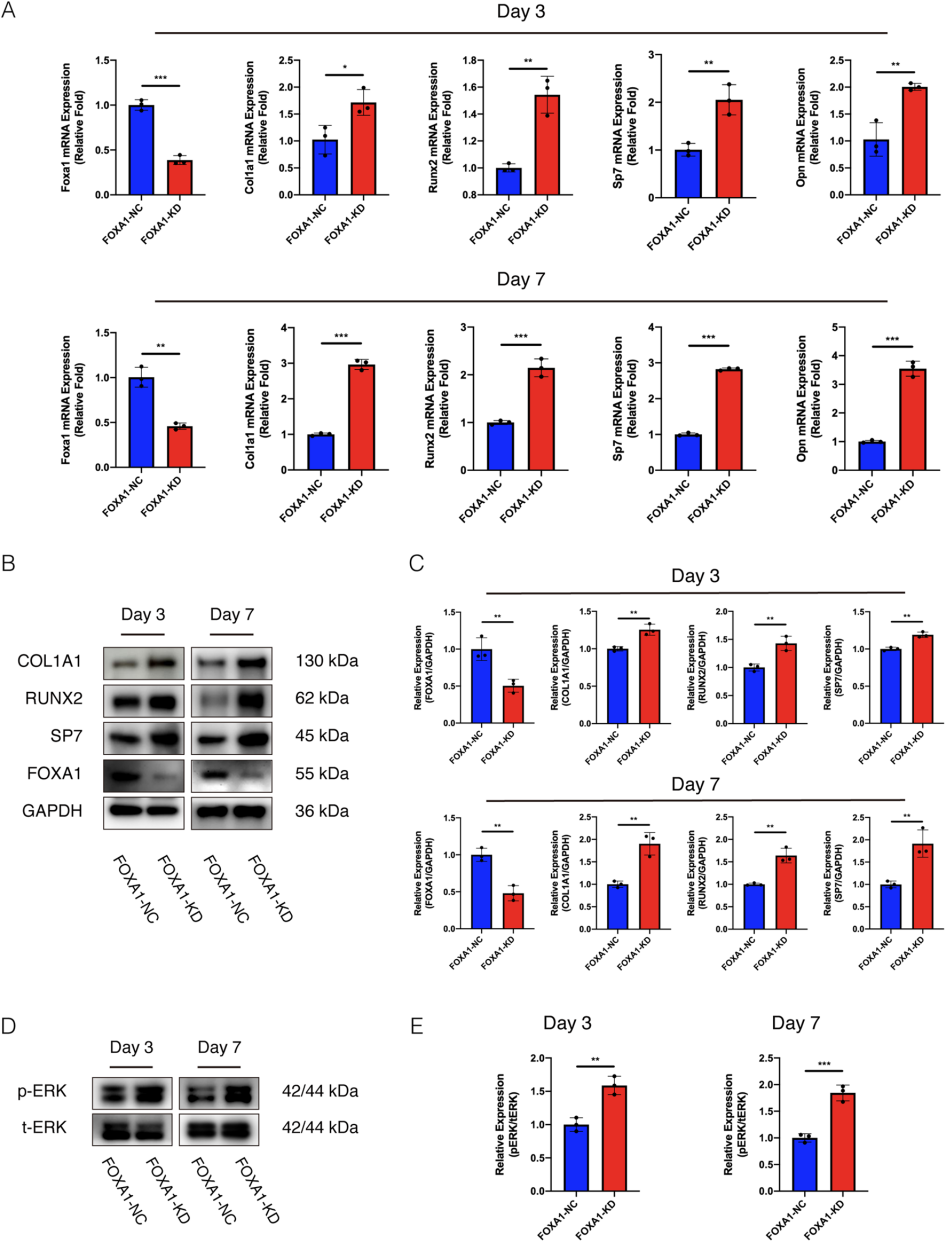
FOXA1 knockdown enhanced the osteo-specific gene and protein levels, activated the ERK1/2 signalling pathway. **A** The mRNA expression levels (normalised to that of 18S) of FOXA1, COL1A1, RUNX2, SP7, OPN in FOXA1-NC and FOXA1-KD groups after 3 and 7 days of osteogenesis. **B**, **C** The protein expression levels (normalised to that of GAPDH) of FOXA1, COL1A1, RUNX2 and SP7 in FOXA1-NC and FOXA1-KD groups after 3 and 7 days of osteogenesis. **D**, **E** The level of p-ERK1/2 (normalised to that of ERK1/2) increased after 3 and 7 days of osteogenesis in FOXA1-KD group. All data are means ± SDs (*n* = 3). *p < 0.05, **p < 0.01, and ***p < 0.001 versus the control group

**Fig. 3:**
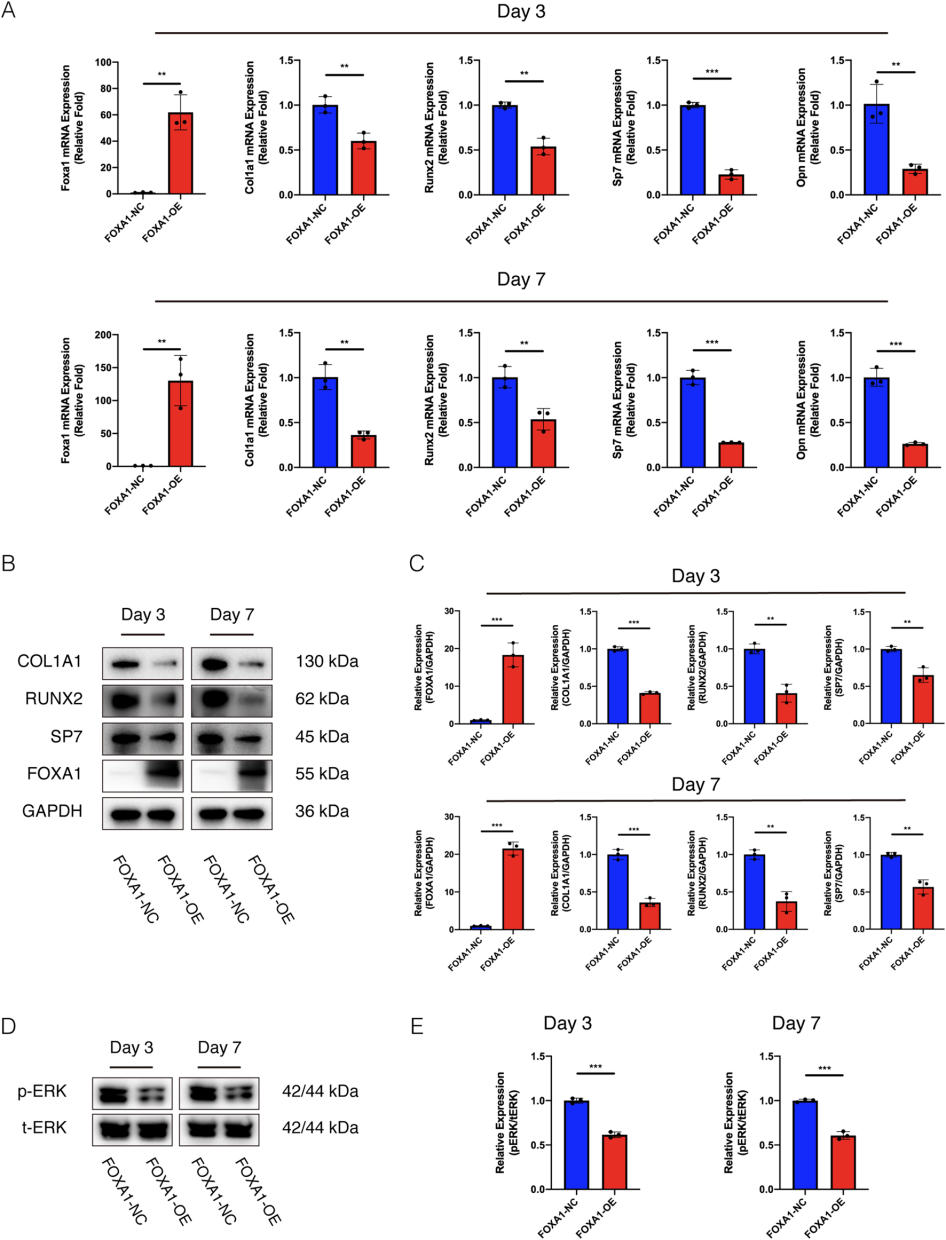
FOXA1 overexpression decreased the osteo-specific gene and protein levels, inhibited the ERK1/2 signalling pathway. **A** The mRNA expression levels (normalised to that of 18S) of FOXA1, COL1A1, RUNX2, SP7, OPN in FOXA1 OE-NC and FOXA1 OE groups after 3 and 7 days of osteogenesis. **B**, **C** The protein expression levels (normalised to that of GAPDH) of FOXA1, COL1A1, RUNX2 and SP7 in FOXA1 OE-NC and FOXA1 OE groups after 3 and 7 days of osteogenesis. **D**, **E** The level of p-ERK1/2 (normalised to that of ERK1/2) decreased after 3 and 7 days of osteogenesis in FOXA1-OE group. All data are means ± SDs (*n* = 3). *p < 0.05, **p < 0.01, and ***p < 0.001 versus the control group

To clarify the role of FOXA1 in the osteogenic differentiation of BMSCs, endogenous FOXA1 was efficiently downregulated using a lentiviral vector system. FOXA1 expression was determined using quantitative real-time PCR and western blot analyses with osteogenic induction for 3 and 7 days after infection and puromycin screening. The expression of FOXA1 was significantly decreased in the FOXA1-KD group compared to that in the FOXA1 KD-NC (Fig. 2A, B) and significantly increased in the FOXA1-OE group compared to that in the FOXA1 OE-NC (Fig. 3A,B).

### FOXA1 knockdown and overexpression did not affect hBMSC proliferation

**Fig. 4:**
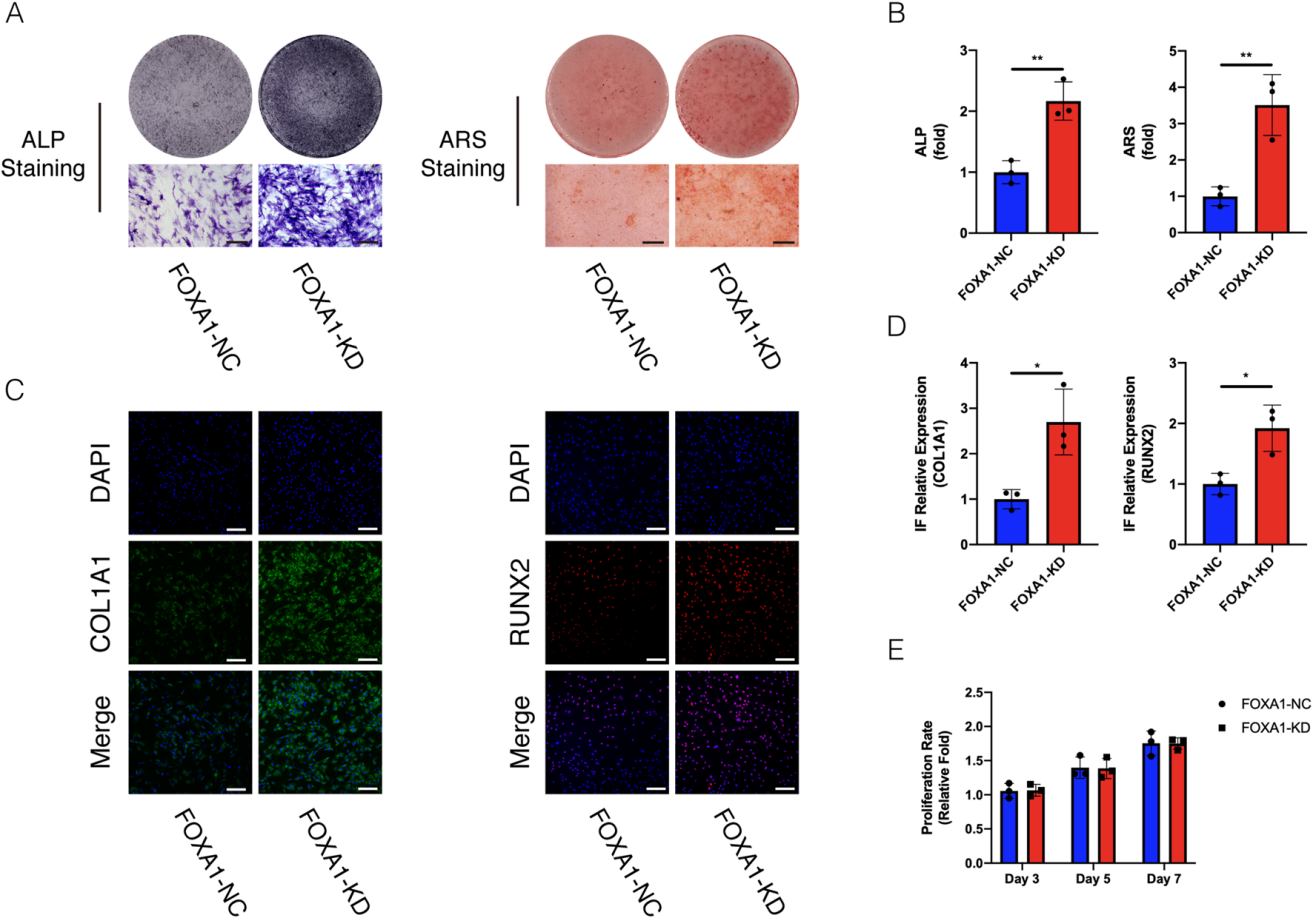
Stain effects of FOXA1 knockdown on osteogenic differentiation and proliferation by FOXA1 knockdown of hBMSCs. **A**, **B** Knockdown of FOXA1 significantly enhanced hBMSC ALP activity (after 7 days of osteogenesis) and calcium deposits. (after 14 days of osteogenesis) in ALP and ARS staining. Scale bars = 50 μm. **C**, **D** Relative expression of osteo-specific proteins (COL1A1 and RUNX2) determined by IF on day 5 of osteogenesis. Nuclei were counterstained with DAPI. Scale bars = 200 μm. **E** The CCK-8 assay revealed that hBMSCs proliferation was not affected by FOXA1 knockdown (FOXA1-KD). All data are means ± SDs (*n* = 3). *p < 0.05, **p < 0.01, and ***p < 0.001 versus the control group

**Fig. 5:**
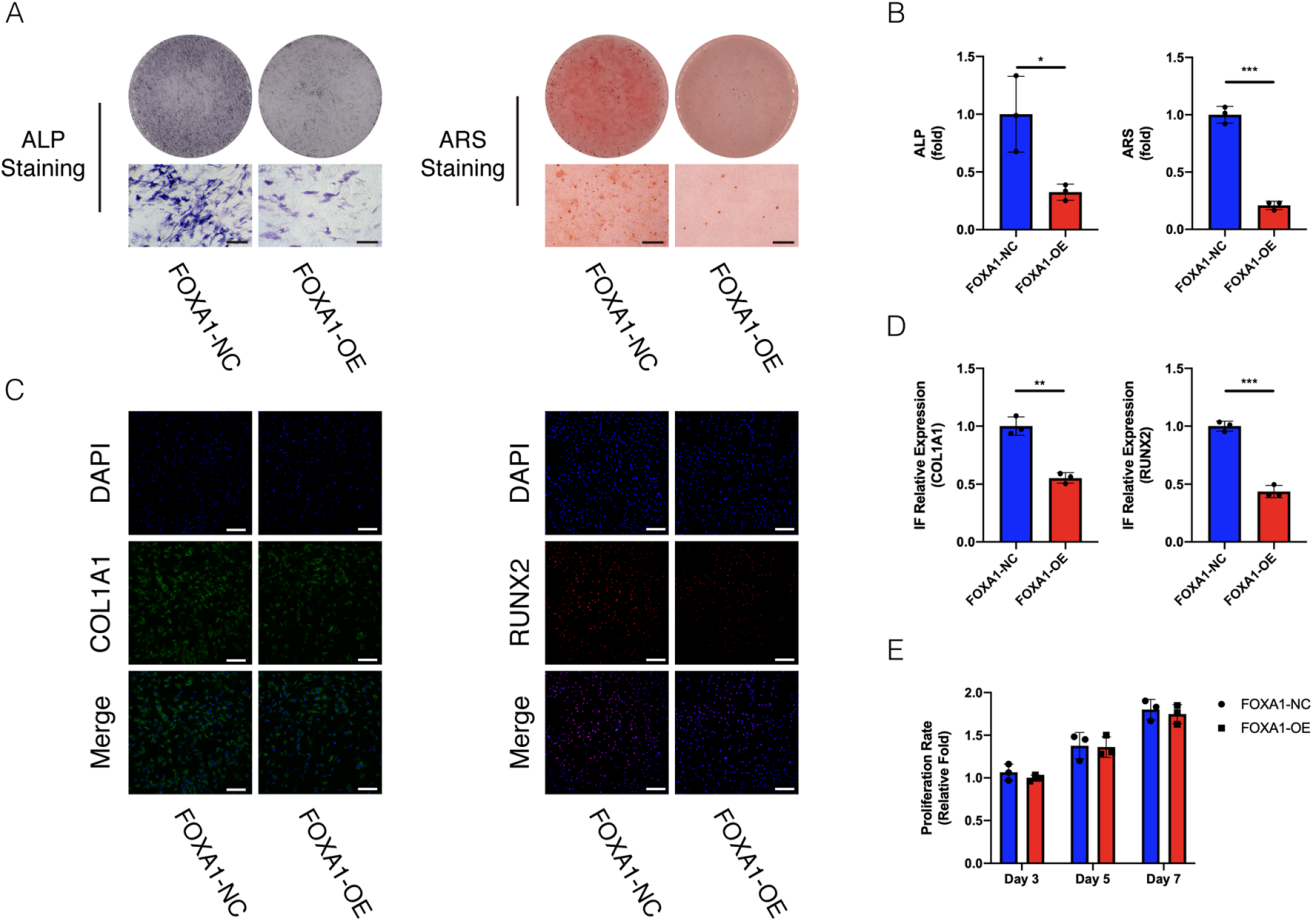
Stain effects of FOXA1 overexpression on osteogenic differentiation and proliferation by FOXA1 overexpression of hBMSCs. **A**, **B** overexpression of FOXA1 significantly reduced hBMSC ALP activity (after 7 days of osteogenesis) and calcium deposits. (after 14 days of osteogenesis) in ALP and ARS staining. Scale bars = 50 μm. **C**, **D** Relative expression of osteo-specific proteins (COL1A1 and RUNX2) determined by IF on day 5 of osteogenesis. Nuclei were counterstained with DAPI. Scale bars = 200 μm. **E** The CCK-8 assay revealed that hBMSCs proliferation was not affected by FOXA1 overexpression (FOXA1-OE). All data are means ± SDs (*n* = 3). *p < 0.05, **p < 0.01, and ***p < 0.001 versus the control group

hBMSC proliferation was analysed on days three, five, and seven following lentiviral particle infection using a Cell Counting Kit-8 (CCK-8) staining. There was no significant difference in the proliferation rate between the FOXA1-KD and KD-NC groups (Fig. 4E), also in the FOXA1 OE and OE-NC groups (Fig. 5E).

### FOXA1 knockdown increased the osteo-specific gene and protein levels and activated the ERK1/2 signalling pathway but FOXA1 overexpression decreased the osteo-specific gene and protein levels

To assess the role of FOXA1 in the osteogenic differentiation of BMSCs, the levels of osteo-specific genes and proteins including RUNX2, SP7, osteocalcin (OCN), COL1A1, and OPN were detected using qRT-PCR and western blotting analysis. On days three and seven, qRT-PCR demonstrated that COL1A1, RUNX2, SP7 and OPN mRNA levels were significantly increased in the FOXA1-KD group compared to the KD-NC group (*p* < 0.05) (Fig. 2A), significantly reduced in the FOXA1OE group compared to the OE-NC group (*p* < 0.05) (Fig. 3A). Moreover, the protein levels of the osteo-specific markers COL1A1, RUNX2 and SP7 were higher in the FOXA1-KD group than in the KD-NC group on days 3 and 7 (Fig. 2B, C) and lower in the FOXA1 OE group than in the OE-NC group (Fig. 3B, C). Furthermore, on day 3, the protein levels of the osteo-specific markers RUNX2 and COL1A1 were higher in the FOXA1-KD group than in the KD-NC group but lower in in the FOXA1 OE group than in the OE-NC group, as evaluated using immunofluorescence.

To explore the signalling pathways involved in the regulation of hBMSC osteogenic differentiation by FOXA1 knockdown, we detected the expression of common signalling pathways using western blotting analysis. p-ERK1/2 levels were significantly increased in the FOXA1-KD group compared with the KD-NC group and significantly decreased in the FOXA1-OE group compared with the OE-NC group, whereas no significant differences were found in the levels of total ERK1/2 (t-ERK1/2) on days 3 and 7 (Figs. 2D, E, 3D, E).

### FOXA1 knockdown enhanced alkaline phosphatase (ALP) activity and calcium deposit formation whereas FOXA1 overexpression decreased ALP activity and calcium deposit formation

ALP activity is a marker of early osteogenesis. During osteogenic differentiation, ALP activity was assessed on day seven. ALP activity was higher in the FOXA1-KD group than in the KD-NC group (*p* < 0.05), and ALP staining showed similar results (Fig. 4A, B). Compared with the FOXA1 OE-NC group, lower ALP activity was observed in the FOXA1 OE group (*p* < 0.05)(Fig. 5A,B).

Calcium deposits were examined using Alizarin Red staining (ARS). More calcium deposits were observed in the FOXA1 knockdown group than in the KD-NC group, whereas less calcium deposits were detected in the FOXA1 OE group than in the OE-NC group on day 14, and quantification analysis showed similar results (Figs. 4A, B, 5A, B).

### Partial rescue by the addition of ERK1/2 signalling inhibitor

**Fig. 6:**
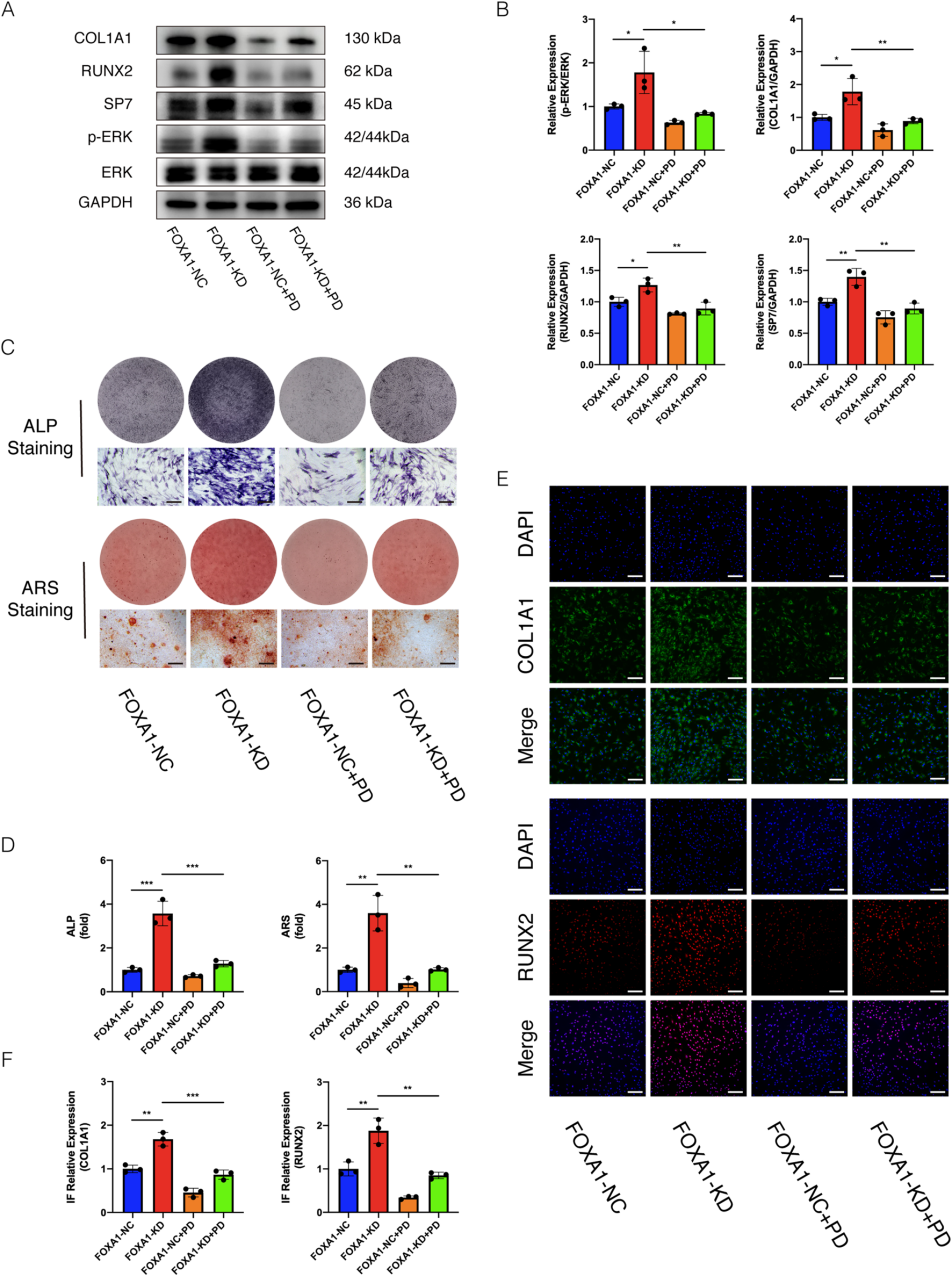
Enhanced osteogenic differentiation of hBMSCs due to FOXA1 knockdown was partially reduced by the addition of ERK1/2 signalling inhibitors. **A**, **B** Increased expression of osteo-specific proteins COL1A1, RUNX2, SP7 (normalised to that of GAPDH) and the level of p-ERK1/2 (normalised to that of ERK) induced by FOXA1 knockdown were almost suppressed by PD98059. **C**, **D** Increased ALP activity and mineralisation of hBMSCs by FOXA1 knockdown were attenuated by PD98059. Scale bars = 100 μm. **E**, **F** The inhibitor almost completely suppressed the increased RUNX2 and COL1A1 proteins detected by IF. All data are means ± SDs (*n* = 3). *p < 0.05, **p < 0.01, and ***p < 0.001 versus the control group

To further confirm the participation of the ERK1/2 signalling pathway in osteogenesis in the FOA1-KD group, we used PD98059 (40 umol/ml), an effective inhibitor of the ERK1/2 signalling pathway. The addition of the inhibitor for 5 d almost completely abrogated the promotive effect on RUNX2, SP7, and COL1A1 expression induced by FOXA1 knockdown (Fig. 6A, B). Similar results were obtained based on immunofluorescence, and inhibition of ERK1/2 partially reversed the increase in the expression of the osteo-specific genes RUNX2 and COL1A1 (Fig. 6E, F). Moreover, the level of p-ERK1/2 was significantly lower than in the FOXA1 KD group without the inhibitor (Fig. 6A, B). Furthermore, ALP activity and staining showed higher ALP activity in FOXA1 knockdown hBMSCs than in the FOXA1 KD + inhibitor group. In addition, ARS assay indicated that the increase in calcium deposits by FOXA1 knockdown was suppressed by PD98059 (Fig. 6C, D).

### mBMSCs with FOXA1 knockdown show accelerated bone healing in a mouse model

**Fig. 7:**
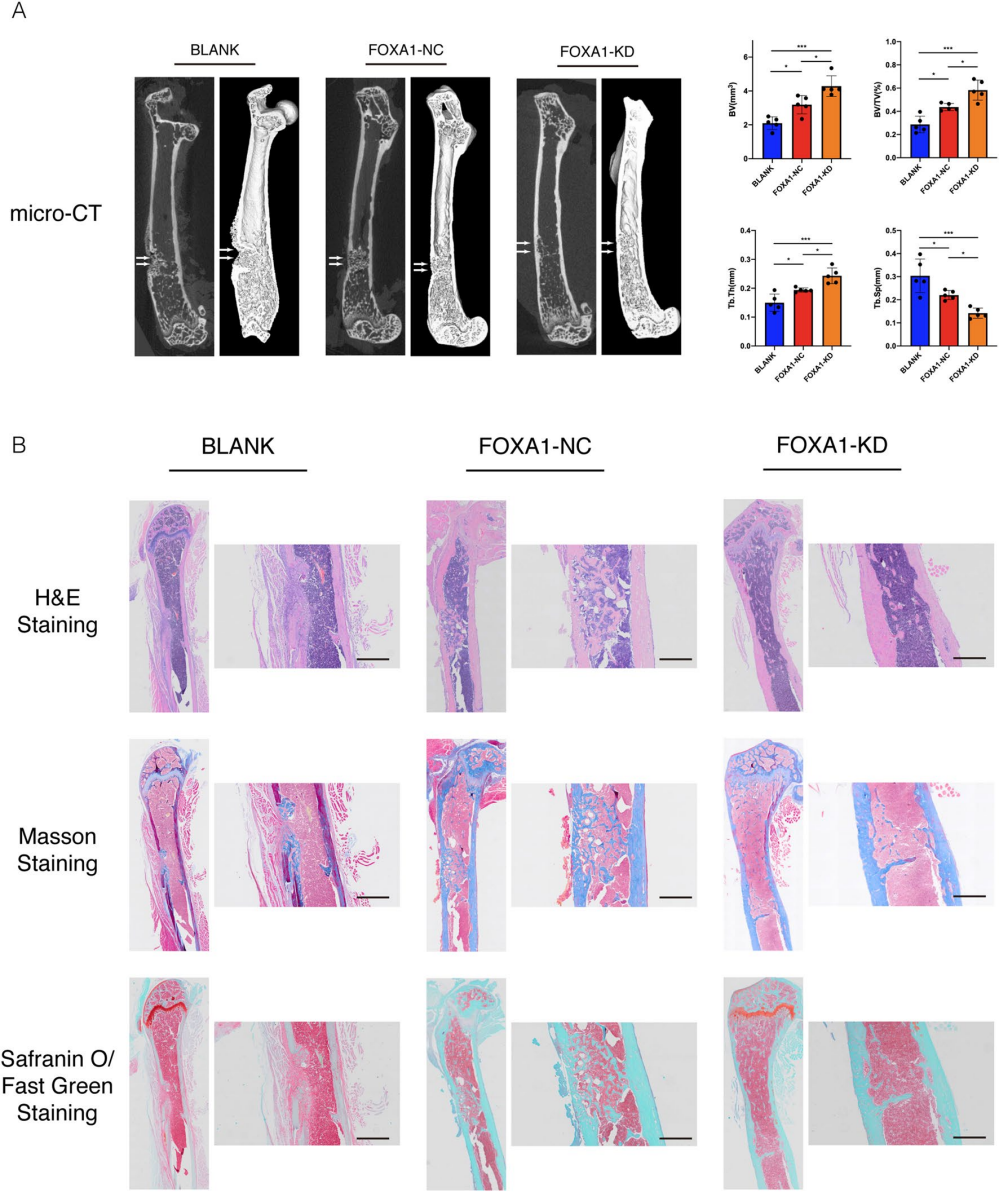
A sheet of mBMSCs with FOXA1 knockdown accelerated bone healing in a mouse model of femoral defects. **A** Microcomputed tomography analysis for bone healing. The sheet with FOXA1 knockdown increased the BV, BV/TV and Tb.Th and decreased the Tb.Sp (*n* = 5). **B** Histological analysis for bone healing. Hematoxylin and Eosin (H&E) staining, Masson’s Trichrome staining and Safranin O/Fast Green staining, Scale bars = 500 μm. White arrow: the defect area. All data are means ± SDs. *p < 0.05, **p < 0.01, and ***p < 0.001 versus the control group

Sheets of mBMSCs with FOXA1 knockdown were used in a mouse femoral defect model to further examine the effect of FOXA1-KD in vivo. Based on microcomputed tomography (microCT) 4 weeks after surgery, the cortical defects on the images were clearly visible in the blank group. This gap was narrower in the KD-NC group than in the blank group, and bridging callus formation was detected. Compared with the other two groups, the defects had nearly disappeared in the KD group, and more bridging callus formation was observed (Fig. 7A). Histological analysis of bone regeneration, including haematoxylin and eosin (HE), safranin O/fast green, and Masson's trichrome staining, revealed that the defects in the blank group were filled with fibrous tissue, although no bridging bone formation was detected. Some calluses containing newly formed woven bone tissue were present at the defect sites of the KD-NC group. In contrast to the other two groups, the defect locations were almost entirely healed, and callus reconstruction was more comprehensive in the FOXA1 knockdown group (Fig. 7B).

## Discussion

Currently, a great number of studies have concentrated on the identification of biomarkers using differential expression analysis, which is a widely used approach to identify candidate genes in the first step of screening. However, almost all of these studies have concentrated on a single experimental group. In this study, two experimental groups (i.e. osteogenically induced samples with hDPSC and hBMSC sources) were used to identify differentially expressed miRNAs that could significantly improve the reliability of candidate miRNAs in the regulation of osteogenic differentiation. Based on the results, 22 miRNAs were identified as candidate genes associated with osteogenic differentiation. Among these, most were downregulated during osteogenic differentiation, suggesting that the downregulation of these miRNAs can significantly promote ossification by directly targeting osteogenic differentiation-related pathways. Therefore, the target genes that are directly regulated by these miRNAs are potential osteogenic differentiation-related drivers and deserve further investigation to determine their role in the regulation of osteogenic differentiation.

As shown in Table 2, we found that 22 differentially expressed miRNAs are shared between DPSCs and BMSCs during osteogenic differentiation. This is different from the results of the study by Gaus et al. [69], in which 16 overlapped miRNAs are identified to be differentially expressed. The study by Gaus et al. made use of different packages (‘limma’ and ‘DEseq2’) and a *p* value threshold (*p* < 0.05) in their differential expression analysis. In contrast, for consistency, we used only ‘limma’ to identify differentially expressed miRNAs, and a threshold of adjusted *p* value < 0.05 to search for candidate miRNAs. However, the miRNAs overlapping in the study by Gaus et al. tended to be differentially expressed, in agreement with our analysis. If we reduced the threshold of fold change, these miRNAs would be considered differentially expressed in our analysis. For the analysis of principal transcription factors, Gaus et al. calculated the difference in degree, average path length, betweenness centrality, closeness centrality, and the topological coefficient for each transcription factor in the miRNA-TF network. In contrast, we calculate the targeted degree of each transcription factor to identify significant biomarkers in the network and rank them based on their interactions and select those with the most interactions as candidate biomarkers of osteogenic differentiation. Although the detailed analysis—including packages, threshold, parameters, and pre-processing—is different between our study and that of Gaus et al., almost all transcription factors identified by us were also presented by Gaus et al., and the transcription factor, FOXA1, was commonly identified as the most significant and novel osteogenic differentiation biomarker. These similar results further demonstrate the reliability of our study, meaning both studies provide significant and reliable results on osteogenic differentiation-related biomarkers. Therefore, we propose that these candidate biomarkers are worthy of further investigation in future research.

FOXA1 was identified as the key transcription factor in the osteogenic differentiation of hBMSCs, which is directly targeted by 12 miRNAs in our study and with the highest degree of difference in the study by Gaus et al. Therefore, a FOXA1-KD or OE strategy was used to explore the role that FOXA1 plays in the osteogenic differentiation of hBMSCs. This showed that FOXA1-KD accelerated osteogenesis in hBMSCs with the activation of the ERK1/2 signalling pathway in vitro. Moreover, an mBMSC sheet with knocked down FOXA1 accelerated bone fracture healing in a mouse femoral defect model. These findings convincingly indicate that FOXA1-KD enhances the osteogenic differentiation of hBMSCs, at least partially, by activating the ERK1/2 signalling pathway.

The results of this study clearly demonstrate the novel and pivotal role of FOXA1 in regulating the osteogenesis of hBMSCs. The FOX family is a group of transcription factors containing the ‘Forkhead’ motif [70], which plays an regulatory role in bone metabolism and orthopaedic diseases [29]. FOXA1, a member of the Forkhead family of winged-helix transcription factors, has been mainly reported for its role in the regulation of development and differentiation in several organs [26–28, 31, 32] as well as its participation in cancer [30, 34, 35]. Previous studies have revealed the dual regulatory role of FOXA1 in Estradiol (E2)-signalling using genome-wide ChIP-chip analysis [30, 71, 72]. In addition, E2 can activate extracellular signal-regulated kinase (ERK) [73, 74], which plays a vital role in the osteogenic differentiation of mesenchymal stem cells [75–77] and has a large effect on skeletal growth, bone maturation ^63^, and bone metabolism [78].

Immunoprecipitation revealed a physical interaction between FOXA1 and AR, which was confirmed by glutathione-S-transferase pull-down assays. This interaction is directly mediated by AR's DNA-binding domain/hinge region and FOXA1's Forkhead domain [31, 79, 80]. Earlier studies have shown that androgens/ARs can modulate the functions of BMSCs to inhibit adipogenesis and promote osteogenesis [66, 81, 82], which also plays a key role in the maintenance of male skeletal integrity [83]. Jin et al. [80] also presented evidence that FOXA1 inhibits AR signalling. Interestingly, these studies are consistent with our results that the knockdown of FOXA1 enhances the osteogenic differentiation of BMSCs.

MEK1/2-ERK1/2 MAPK signalling plays a critical role in regulating the functions of osteoblasts and osteoclasts [84–86]. In this study, FOXA1 knockdown increased the ERK1/2 signalling pathway during osteogenesis. Furthermore, the inhibitor PD98059 blocked ERK1/2 phosphorylation and partially rescued the increase in osteogenesis of hBMSCs induced by FOXA1 knockdown. These findings clearly demonstrated that ERK1/2 plays a crucial role in FOXA1-KD-induced osteogenesis in hBMSCs.

Many members of the FOX gene family play a role in the osteogenic differentiation of BMSCs. This is the first study to demonstrate the effect of FOXA1 on hBMSC osteogenesis. Given the current focus on the impact of FOXA1 knockdown on osteogenesis, the mechanisms of ERK1/2 signalling pathway activation by FOXA1 knockdown are not yet clearly understood, and whether downstream targets act through AR and E2 is also unclear. In future studies, other signalling pathways and downstream targets should be explored for their potential involvement in osteogenesis in hBMSCs via FOXA1 knockdown.

## Conclusions

**Fig. 8:**
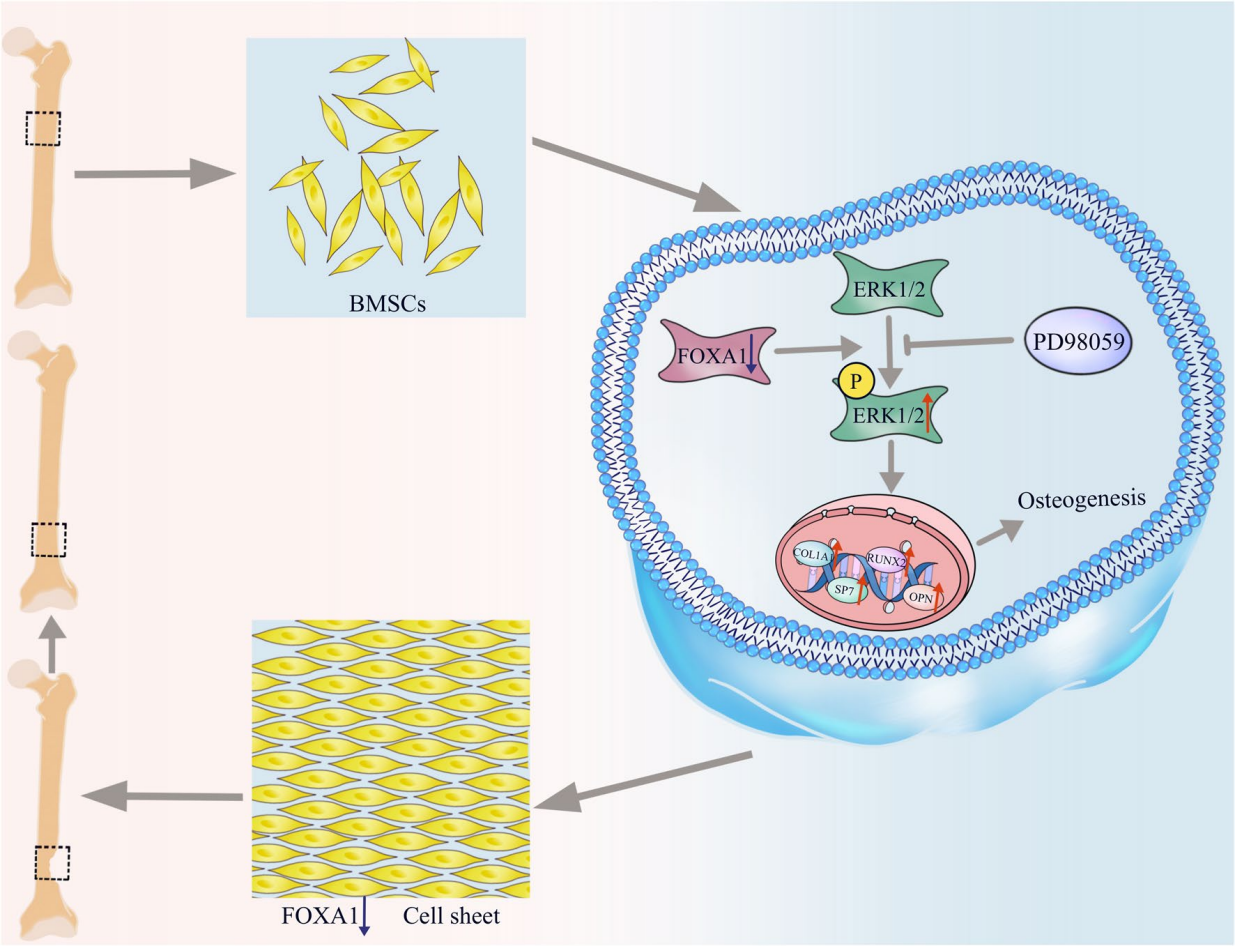
The schematic of the experiment is shown in the figure

FOXA1-KD enhanced osteogenic differentiation of hBMSCs, partly via activation of the ERK1/2 signalling pathway. The mBMSC sheet containing FOXA1-KD effectively promotes healing of bone defects in mice (Fig. 8).

## Data Availability

The datasets used and/or analysed during the current study are available from the corresponding author on reasonable request.

## Abbreviations

hBMSCs: Human bone mesenchymal stem cells
hDPSCs: Human dental pulp stem cells
GAPDH: Glyceraldehyde-3-phosphate dehydrogenase
Osx/SP7: Osterix
OCN: Osteocalcin
ALP: Alkaline phosphatase
ARS: Alizarin Red staining
OPN: Osteopontin
RUNX2: Runt-related transcription factor 2
COL1A1: Collagen type I alpha 1
ERK1/2: Extracellular signal-regulated kinase 1/2 MAPK
p-ERK1/2: Phospho-ERK1/2
IF: Immunofluorescence
GEO: Gene Expression Omnibus
GFP: Green fluorescent protein
qRT-PCR: Quantitative real-time PCR
E2: Estradiol
